# Diagnostic Challenges in Enteropathies: A Histopathological Review

**DOI:** 10.3390/diagnostics15121511

**Published:** 2025-06-13

**Authors:** Iulia Enache, Ioan-Cristian Nedelcu, Marina Balaban, Daniel Vasile Balaban, Alina Popp, Mariana Jinga

**Affiliations:** 1 Internal Medicine and Gastroenterology Department, University of Medicine and Pharmacy “Dr. Carol Davila”, 020021 Bucharest, Romania; iulia.enache@drd.umfcd.ro (I.E.); nedelcu.i.c@gmail.com (I.-C.N.); marina.balaban@yahoo.com (M.B.); vasile.balaban@umfcd.ro (D.V.B.); mariana_jinga@yahoo.com (M.J.); 2 Department of Gastroenterology, Dr. Carol Davila Central Military Emergency University Hospital, 010825 Bucharest, Romania; 3 Department of Pediatrics, University of Medicine and Pharmacy “Dr. Carol Davila”, 020021 Bucharest, Romania; 4 Department of Pediatrics, Alessandrescu-Rusescu National Institute for Mother and Child Health, 020395 Bucharest, Romania; 5 Celiac Disease Research Center, Faculty of Medicine and Health Technology, Tampere University and Tampere Hospital, 33014 Tampere, Finland

**Keywords:** enteropathy histopathology, immune-mediated enteropathy, celiac disease, non-celiac enteropathy, drug-induced enteropathy

## Abstract

Various enteropathies, including immune-mediated (IME) and infection-related conditions, can lead to small intestinal mucosal injury and malabsorption. While immune dysregulation plays a central role in diseases like celiac disease and autoimmune enteropathy, other conditions such as small intestinal bacterial overgrowth (SIBO) and tropical sprue (TS) involve infectious or microbial pathogenesis. Common clinical manifestations include weight loss, chronic diarrhea, and nutritional deficiencies. While celiac disease (CD) remains the most prevalent IME in adults, an expanding spectrum of non-celiac enteropathies has been recognized, including autoimmune enteropathy (AIE), common variable immunodeficiency disease (CVID), olmesartan-induced enteropathy, tropical sprue, and small intestinal bacterial overgrowth. These conditions often present with overlapping clinical, serological, and histological features, complicating their differentiation from CD. Accurate diagnosis is critical for the timely initiation of effective treatment to prevent disease progression and associated complications such as severe malabsorption and enteropathy-associated T-cell lymphoma (EATL). The small intestine plays a dual role in nutrient absorption and immune regulation, making it uniquely vulnerable to immune dysregulation. In IMEs, hyperactive immune responses disrupt intestinal homeostasis, leading to mucosal damage and impaired nutrient absorption. Although CD is the prototypical IME, increasing the recognition of non-celiac IMEs, it highlights the need for a more nuanced approach to small bowel biopsy interpretation. This review explores the histopathological and clinical features of common IMEs, with a focus on distinguishing non-celiac disorders that mimic CD. By enhancing the understanding of these conditions, this review aims to improve diagnostic accuracy, facilitate appropriate therapeutic interventions, and mitigate complications associated with delayed or misdiagnosis. A multidisciplinary approach involving gastroenterologists and pathologists is emphasized to optimize outcomes for patients with IMEs. Immune-mediated enteropathies result from an abnormal immune response of the small intestinal mucosa to non-pathogenic molecules, often leading to malabsorption syndrome. The most common symptoms include weight loss, chronic diarrhea, and nutritional deficiencies. While celiac disease (CD) is the most well-known immune-mediated enteropathy (IME) in adults, other related disorders have been identified in recent years. These conditions share many clinical and histopathological features, therefore making differentiations between them challenging. This study aims to review the most common immune-mediated enteropathies, with a focus on non-celiac disorders that should be considered in the differential diagnosis of celiac disease in small bowel biopsies.

## 1. Introduction

The small intestine serves two main functions: nutrient absorption and immune system regulation. It contains the largest surface area for the efficient absorption of nutrients from ingested food and constitutes the most extensive component of the human immune system. This dual role is critical, given its continuous exposure to various substances from the external environment. The intestinal immune system must distinguish between harmless materials and harmful antigens, responding appropriately by either mounting an immune reaction or developing tolerance, depending on the antigens encountered [1]. A range of enteropathies can disrupt both of these functions, either through immune-mediated mechanisms—as seen in conditions like celiac disease or autoimmune enteropathy—or through infectious and microbiota-related processes, such as those implicated in SIBO, tropical sprue, or common variable immunodeficiency (CVID). These diverse pathogenic mechanisms can ultimately result in similar outcomes such as mucosal injury and malabsorption syndromes. A thorough understanding of the intricate relationship between the small intestine and the immune system is essential for the diagnosis and management of various gastrointestinal disorders.

Many enteropathies, whether immune-mediated or of infectious and idiopathic origin, often present overlapping clinical, serological, and histological features, complicating their differentiation. Early recognition of these enteropathies is vital for initiating prompt and effective treatment, which can help prevent disease progression and its associated complications, notably severe malabsorption. Malabsorption can lead to nutrient deficiencies and other health issues. A particularly concerning complication is the heightened risk of enteropathy-associated T-cell lymphoma (EATL). Individuals with conditions such as celiac disease (CD), refractory CD, or autoimmune enteropathy (AIE) face an increased risk of developing this aggressive form of lymphoma [2].

Celiac disease is highlighted as the most common immune-mediated enteropathy in adults [3]. However, the diagnostic landscape has broadened considerably in recent years, encompassing a diverse array of small intestinal disorders. These include autoimmune conditions such as autoimmune enteropathy (AIE), immune deficiency, associated conditions like common variable immunodeficiency disease (CVID), drug-induced enteropathies (e.g., olmesartan-associated enteropathy), and infectious or microbiota-related disorders, such as tropical sprue and small intestinal bacterial overgrowth (SIBO). Although these conditions differ in their underlying mechanisms, they may produce overlapping histological changes and similar clinical presentations, necessitating careful diagnostic differentiation.

## 2. Methods

For the purpose of this review, we conducted a search of the literature for published manuscripts on immune-mediated enteropathies. The search was performed in March 2024 on the PubMed (MEDLINE) database. The following keywords were searched for: “immune-mediated enteropathy histopathology”; “auto-immune enteropathy histopathology”; “olmesartan enteropathy histopathology”; “non-celiac villous atrophy histopathology”; “celiac-like enteropathy histopathology”; “protein-losing enteropathy histopathology”; “HIV enteropathy histopathology”; and “common variable immunodeficiency (CVID) enteropathy histopathology”.

The search was restricted to full-text English-language articles on adults, published in the last 10 years (2014–2024) and excluded case reports, letters, and papers referring to animal studies or children.

We screened titles according to relevance to the researched topic. Among the immune-mediated enteropathies, we included celiac disease, auto-immune enteropathies, drug-induced enteropathies, infectious enteropathies (tropical sprue), CVID enteropathy and SIBO. From a total of 863 papers, we selected a final 50 for analysis. In addition to the primary search, older studies published before 2014 were included when repeatedly cited in the more recent literature, particularly when they provided foundational classifications or pathologic descriptions.

## 3. Normal Histology Findings in the Duodenum

The architecture of the duodenal villi is influenced by biopsy depth. When the muscularis mucosae is present, the villi are typically taller and slenderer, with glands that are orderly arranged and regularly spaced, resting directly on the muscularis mucosae. Normally, the villi length can reach five times the crypt depth, though this ratio usually is 3:1, with some structural differences in the proximal duodenum (duodenal bulb), where villi are shorter [4,5]. The villi are covered by a columnar epithelium, consisting primarily of enterocytes with a brush border. A shift in the morphology of enterocytes from columnar to cuboidal is indicative of injury. The intestinal epithelium is composed of a mixture of enterocytes, goblet cells, sparsely interspersed between enterocytes and responsible for mucus secretion, and Paneth cells, which are exocrine cells that secrete lysozyme and are typically located at the base of the crypts. Occasionally, enterochromaffin cells may also be found within the crypts. In the duodenal bulb, the villi tend to be shorter and broader, often exhibiting branching, a variation in normal histology that is attributed to increased acidity in this region [6].

Intraepithelial lymphocytes (IELs) are consistently present in the duodenal epithelium, distributed along the sides of the villi. However, the normal range for IEL counts has been a subject of debate. Some studies have reported a typical IEL count of approximately 10 IELs per 100 epithelial cells [7], whereas others have proposed an upper limit of 25 lymphocytes across five high-magnification fields [8,9]. Consequently, counts of 25–30 IELs per 100 enterocytes are considered borderline, while values exceeding 30 IELs per 100 enterocytes are regarded as pathological [10,11,12]. However, regarding celiac disease, an IEL count greater than 25 per 100 enterocytes is generally considered abnormal and supportive of CD, particularly in the appropriate clinical and serological context [13]. Another important characteristic of IELs is their greater density in the duodenal bulb compared to the distal duodenum, necessitating the reporting of a mean value between the two regions [14]. IELs are part of the gut-associated lymphoid tissue (GALT), which includes lymphoid follicles, Peyer’s patches, and mesenteric lymph nodes. They are primarily T-lymphocytes that co-express CD3 and CD8 antigens [15]. This is clinically significant, as immunohistochemical staining, such as CD3 immunostaining, can enhance the accuracy of IELs detection. In cases where duodenal biopsies reveal a borderline increase in IELs using conventional staining methods, such as hematoxylin and eosin, CD3 immunostaining should be employed to determine whether the IEL count is within normal limits or indicative of pathology [16]. In normal duodenal mucosa, IELs exhibit a “decrescendo” pattern, with fewer IELs at the villi tips than in the proximal third of the villi. Clusters of IELs at the villi tips are always considered abnormal [17].

An increase in IELs with preserved villous architecture is a common finding in duodenal biopsies (Table 1) [10,18]. Studies have shown that only 20% of biopsies with increased IELs and normal villous architecture confirm a diagnosis of celiac disease [18,19].

The lamina propria of normal duodenal mucosa is predominantly populated by plasma cells, with smaller numbers of lymphocytes, eosinophils, mast cells, and mesenchymal elements. Histiocytes and apoptotic bodies are rare. While eosinophils are consistently present, their density varies; one Japanese study reported a mean of 33.51 eosinophils/mm^2^ (range 12.64 to 56.44/mm^2^) in adults [20]. The presence of granulocytes is considered normal, provided they are not found in the crypts or surface epithelium. Subepithelial collagen should be thin, measuring between 3 and 5 µm, and evenly distributed.

Brunner’s glands, which are submucosal glands responsible for secreting alkaline mucus to protect the duodenal lining from gastric acid, are a notable feature in bulb biopsies. These glands are located on both sides of and between elements of the muscularis mucosae. Hyperplasia of Brunner’s glands is associated with broader, shorter villi, and the villous height-to-crypt depth ratio in this region is considered normal at 2:1 [21]. Duodenal biopsies typically terminate at the submucosa.

Increased IELs accompanied by progressive hyperplasia of the crypts and villous atrophy is usually suggestive of celiac disease, although other conditions should be considered (Table 1). Regardless of biopsy location, it is crucial to evaluate mucosal histology using high-quality, well-oriented, and precisely cut samples to avoid misclassification and misdiagnosis [22,23].

### 3.1. Celiac Disease (CD)

Celiac disease (CD) is an immune-mediated enteropathy triggered by dietary gluten in genetically predisposed individuals. The estimated global prevalence of CD is approximately 1.4%, with a higher incidence observed in females [24]. Diagnosis of CD in adults is based on a combination of clinical, serological, and histopathological criteria. Although CD is classically associated with malabsorption syndrome, atypical forms presenting with extra-intestinal symptoms, or even asymptomatic cases are increasingly recognized [25,26]. This variability in presentation often leads to long delays in diagnosis. The only effective treatment for CD is a lifelong strict gluten-free diet (GFD), which alleviates clinical symptoms and promotes mucosal healing [27].

CD primarily affects the small intestinal mucosa, where gluten ingestion elicits an abnormal immune response. In CD, intraepithelial lymphocytes (IELs) typically exceed 30 IELs per 100 enterocytes [7,28]. These IELs tend to populate both the tips and sides of the villi and may cluster around the villi tips. The number of IELs correlates with the severity of mucosal atrophy [29]. However, cases of celiac disease with normal IELs densities in the epithelium were reported [30]. The IELs in the duodenum are primary T cells expressing CD3 and CD8 antigens, a feature that can be detected through immunohistochemistry, particularly when evaluating cases of refractory CD [31]. In CD, the enterocytes exhibit morphological changes, with associated mucin depletion and intracytoplasmic vacuolization, indicating a reactive appearance [32]. Increased cellularity in the lamina propria is another characteristic feature, with a proliferation of plasma cells, lymphocytes, eosinophils, histiocytes, and mast cells. Although neutrophils are not typically associated with CD, they may be present in some cases, along with crypt abscesses [33]. Crypt apoptosis, while subtle, is another important histologic finding and can also be observed in autoimmune enteropathy, graft-versus-host disease (GVHD), and mycophenolate toxicity [34]. The subepithelial collagen band in CD should remain thin.

In addition to increased IELs, common mucosal abnormalities in CD include crypt hyperplasia and villous atrophy. Based on these alterations, the Marsh and modified Marsh–Oberhuber criteria were developed to classify the severity of CD-related histological changes [35,36]. It is important to note, however, that villous atrophy is not pathognomonic for CD, as it represents the most severe stage of progressive villous damage, which typically takes years to develop. Corazza and colleagues later proposed a simplified histological classification, categorizing CD lesions into non-atrophic (grade A) and atrophic (grade B) stages. Grade A is defined by an isolated increase in IELs (≥25 per 100 enterocytes), whereas grade B is subdivided into B1, where the villous/crypt ratio is less than 3:1 but villi are still identifiable, and B2, where the villi are completely absent. This classification system has demonstrated reduced variability and improved inter-observer agreement among pathologists [37] (Table 2). For an accurate assessment of villous blunting, it is recommended to identify 3–4 well-oriented villi in a row and carefully evaluate their architecture [5].

CD-related mucosal involvement can be patchy, sometimes detectable only in specific regions, such as the duodenal bulb [38]. The significance of duodenal bulb biopsies was highlighted in a study on ultrashort CD, where the disease was confined to the bulb [39]. However, interpreting bulb biopsies can be challenging due to the presence of Brunner’s glands and lymphoid follicles in the bulb, as well as the high gastric acid load in this region [38]. Therefore, multiple biopsies from different duodenal regions are recommended when CD is suspected. Current guidelines advocate for obtaining 1–2 biopsies from the duodenal bulb and 4 from the distal duodenum [13]. For routine diagnoses, group classifications (e.g., Marsh–Oberhuber, Corazza–Villanacci) are usually used. However, in drug trial outcomes, a more precise standard operating procedure using quantitative histomorphometry has been used [22,40,41,42].

Mucosal healing is the primary goal of CD treatment. It has been suggested that complete recovery may take longer than 12 months of strict adherence to a GFD [43], with one U.S. study indicating that a median recovery time of 3 years may be required in adults [44]. While some guidelines recommend repeating the duodenal biopsy after 1–2 years of a gluten-free diet to confirm mucosal healing, this practice is debated. Increasingly, follow-up biopsies are reserved for patients with persistent symptoms, suspected non-adherence, or at risk of complications, as reflected in recent evidence and guidelines [13,45,46,47].

While the majority of individuals with CD exhibit clinical and histological improvements, following the initiation of a GFD, up to 30% continue to experience persistent symptoms and/or ongoing intestinal inflammation. These patients are classified as having non-responsive celiac disease (NRCD), which may result from dietary non-compliance, delayed mucosal healing, refractory coeliac disease, or an alternative diagnosis. NRCD is generally defined as the persistence of symptoms, clinical signs, laboratory abnormalities, or histopathological features characteristic of CD, despite a minimum of 6–12 months of presumed strict adherence to a GFD. The condition can be further subclassified into primary NRCD, in which there is no initial clinical response to a GFD, and secondary NRCD, where patients initially experience symptom resolution but subsequently develop recurrent symptoms despite continued dietary adherence [48].

### 3.2. Refractory Celiac Disease (RCD)

RCD is diagnosed when symptoms and histologic abnormalities persist despite at least 12 months of a strict gluten-free diet, and after exclusion of ongoing gluten exposure, typically with dietitian support and negative celiac serology [49,50]. On duodenal biopsies, there are still present severe villous atrophy and increased IELs, despite strict adherence to a GFD. In addition, one can also find extensive basal plasmacytosis and subepithelial collagenous thickening (>10 μm), suggesting collagenous sprue as a complication of RCD [51].

RCD may be divided into two types based on the IEL phenotype [52]. RCD I is characterized by a normal IEL immunophenotype, IELs that are positive for both CD3 and CD8 antigens. RCD II has an abnormal population of IELs, characterized by the lack of surface expression of CD3 and of CD8 antigens, but contains intracellular CD3ε (cytoplasmic expression) and a clonal rearrangement of the gamma chain of T-cell receptor (TCRγ) [53,54,55]. This distinction between RCD I and RCD II is important due to the risk of developing enteropathy-associated T-cell lymphoma (EATL), which is greater for RCD II—approximately half of these patients develop EATL within 5 years, than RCI I (less than 14%) [52,56,57,58].

Unlike classical CD, refractory celiac disease is almost invariably symptomatic. RCD I closely resembles active CD, necessitating a diagnosis based on negative celiac serology and confirmation of strict adherence to a GFD by a dietitian. In contrast, RCD II is typically associated with severe malnutrition, often due to ulcerative jejunitis. Extensive ulcers that develop in the jejunum, can result in protein-losing enteropathy, leading to severe diarrhea and secondary hypoalbuminemia. The diagnosis of RCD II requires the identification of a clonal population of abnormal IELs. Although strict adherence to a GFD remains fundamental in the management of RCD, additional therapeutic interventions are often required. One such treatment is open-capsule budesonide, which has demonstrated efficacy in inducing clinical remission and promoting villous recovery in nearly 90% of cases of both RCD I and RCD II. Beyond budesonide, immunosuppressive agents have been employed in RCD I, while chemotherapy with purine analogs has been utilized in the management of RCD II [59].

### 3.3. Enteropathy-Associated T-Cell Lymphoma (EATL)

Enteropathy-associated T-cell lymphoma (EATL) typically arises as a complication of celiac disease; however, it may also occur in conjunction with autoimmune enteropathy [60,61,62,63]. This malignancy predominantly affects the small intestine, leading to clinical manifestations such as recurrent diarrhea, non-responsiveness to a gluten-free diet (GFD), and systemic symptoms including fever and weight loss.

Histologically, EATL is characterized by complete villous atrophy and crypt hyperplasia, accompanied by a significant cellular infiltrate within the lamina propria. Notably, the quantity of plasma cells is markedly increased compared to that observed in celiac disease. The infiltrating cells display a diverse range of cytomorphologic characteristics, indicating a malignant behavior, and exhibit tendencies to invade glandular epithelium and extend beyond the muscularis mucosae.

The World Health Organization (WHO) classification of 2008 delineates two subtypes of EATL: type 1 and type 2, which are differentiated based on their histological features [64]. EATL type 1 is characterized by a heterogeneous histological pattern, which may include either a monomorphic infiltration of medium to large lymphocytes with irregular nuclear contours and variable cytoplasmic content or the presence of large anaplastic lymphocytes. Additionally, EATL type 1 may exhibit significant polymorphism, with varying quantities of eosinophils, histiocytes, small lymphocytes, and plasma cells. This subtype is frequently associated with celiac disease and is prevalent in Europe. Conversely, EATL type 2 is characterized by monomorphic small to medium-sized lymphocytes with round, hyperchromatic nuclei that display a granular chromatin pattern. This subtype is more commonly found in Asia and may also be associated with celiac disease [65]. Importantly, this classification has prognostic and therapeutic implications. Type II tends to follow a more aggressive clinical course, often with poorer response to chemotherapy and worse overall survival, compared to Type I. Therefore, accurate histologic and immunophenotypic subtyping is essential not only for diagnosis but also for guiding management and assessing prognosis.

### 3.4. Collagenous Sprue (CS)

The term “collagenous sprue” (CS) was first introduced by Weinstein et al. in a case involving a patient with celiac disease who initially exhibited a positive response to a gluten-free diet but subsequently experienced a relapse [66]. Several studies with small cohorts (19 and 22 cases, respectively) have indicated that some cases of CS present with a history of gluten-sensitive disease, which may resemble refractory sprue, suggesting the development of CS, a complication of celiac disease. Conversely, other cases demonstrate that individuals do not respond to gluten-exclusion diets upon initial diagnosis, suggesting the presence of de novo collagenous sprue [51,67].

Histological evaluations reveal several features common to celiac disease, including severe villous atrophy, crypt hyperplasia, and an increased number of intraepithelial lymphocytes (IELs). Additionally, a significant deposition of collagen in the subepithelial zone of the lamina propria, typically exceeding 10 μm in thickness and being unevenly distributed, is also seen in collagenous sprue. The count of IELs can vary significantly (ranging from 7 to 92 IELs per 100 epithelial cells) and is often associated with a chronic inflammatory infiltrate in the lamina propria. However, studies have also reported instances of normal IEL counts (fewer than 25 IELs per 100 epithelial cells), which may represent a key histological distinction from celiac disease [51,67]. Additionally, collagenous sprue may be associated with collagenous gastritis, collagenous colitis, or other immune-mediated disorders.

### 3.5. Tropical Sprue (TS)

Malabsorption syndrome, resulting from chronic diarrhea, is often observed in patients who travel to or reside in tropical regions, such as Central America and Southeast Asia [68]. The etiology is presumed to be infectious; however, the specific responsible pathogen has yet to be identified [69]. Although tropical sprue (TS) is not commonly diagnosed in the United States and Europe, it is estimated to account for up to 40% of malabsorption cases in Southern Asia [70]. Unlike celiac disease, TS does not respond to a gluten-free diet but does improve with antibiotic treatment. The condition can evolve in two ways: an acute form affecting individuals who travel to tropical regions and a chronic form affecting long-term residents. The acute phase typically presents symptoms such as acute diarrhea, abdominal pain, fever, myalgia, and weakness. In contrast, the chronic phase is characterized by persistent diarrhea and malabsorption syndrome, which can lead to deficiencies in folate and vitamin B12. Notably, these deficiencies may take up to four years to develop [71].

Histologically, no specific features are typically identified in the duodenum of patients with TS. It often presents in a patchy distribution [72] and, in comparison to celiac disease, individuals with TS generally exhibit less severe villous abnormalities (classified as Marsh–Oberhuber stage 3a), with an increase in IELs that are more frequently observed infiltrating the basal third of the villi or the mucosal crypts. Furthermore, the density of eosinophils in the lamina propria of patients with TS tends to be higher than that seen in patients with celiac disease [73]. The distribution of the disease also differs between celiac disease and TS: celiac disease predominantly affects the proximal small intestine, resulting in iron deficiency anemia, while TS involves the entire small intestine, including the terminal ileum, leading to deficiencies in vitamin B12 and folate. When these deficiencies occur, histopathological reports may indicate megaloblastic changes in the enterocyte nuclei, characterized by enlarged nuclei with decreased mitotic activity. The most significant histological indicator for diagnosing TS would be an ileal biopsy revealing villous atrophy and increased IELs. Additionally, the patient’s travel history serves as a crucial factor in considering this diagnosis.

### 3.6. Common Variable Immunodeficiency (CVID)

Common variable immunodeficiency (CVID) is characterized by inadequate immunoglobulin production, resulting from impaired maturation of B cells into plasma cells. It represents the most prevalent primary immunodeficiency disorder, with an estimated prevalence ranging from 1 in 25,000 to 1 in 50,000 individuals [74]. Chronic diarrhea is the most frequently observed gastrointestinal manifestation in these patients [75]. Recurrent infections are a hallmark of CVID, with Giardia lamblia being the most common gastrointestinal pathogen. This infection can lead to duodenal villous atrophy, subsequently causing malabsorption. Nevertheless, the precise pathophysiological mechanisms underlying villous atrophy in CVID remain unclear, as eradication of the pathogen does not consistently result in clinical and mucosal recovery [76].

In addition to parasitic infections, viral infections, such as Norovirus, have also been identified in patients with CVID. Notably, Norovirus was detected in eight CVID patients exhibiting concurrent villous atrophy, where symptom resolution and histological improvement were observed following viral clearance [77]. A prospective cross-sectional study of 103 CVID patients revealed that the most prevalent histopathological findings included increased intraepithelial lymphocytes (IELs), a severely diminished plasma cell count, and lymphoid hyperplasia. Notably, villous atrophy was observed in only one patient who also presented with elevated IELs (>25 IELs/100 enterocytes). The study further conducted gene-expression microarray analyses and HLA typing among CVID patients with increased IELs, CVID patients with normal IEL counts, untreated celiac disease (CD) patients, and healthy controls. The findings strongly support that CD and CVID with increased IELs are distinct conditions. Additionally, the study cohort did not exhibit chronic Norovirus infections [78].

Differentiating CVID from CD remains particularly challenging, as CVID patients may present with villous atrophy and a moderate increase in IELs. A key distinguishing feature of CVID is the marked depletion of plasma cells within the lamina propria, in contrast to CD. Additional histological findings in CVID may include lymphoid hyperplasia, enhanced crypt apoptosis, elevated intraepithelial neutrophils, and occasionally, small mucosal granulomas. Infections, most commonly giardiasis or cytomegalovirus, are frequently encountered in these cases [78,79,80].

Due to the impaired humoral immune response in CVID patients, they often lack the ability to produce antibodies of any class, including those typically associated with CD. However, false-positive CD-related antibodies may be detected in CVID patients undergoing treatment with human immunoglobulins [81]. While HLA genotyping can assist in excluding CD, a positive result necessitates the absence of histological improvement on a gluten-free diet (GFD) as the sole criterion for excluding CD.

### 3.7. Auto-Immune Enteropathy (AIE)

Autoimmune enteropathy (AIE) is a rare syndrome characterized by chronic diarrhea and unintentional weight loss, arising from immune-mediated damage to the intestinal mucosa. Its estimated incidence is less than 1 in 100,000 individuals. While AIE predominantly affects children, sporadic cases in adults have been reported [82,83,84].

Inherited forms of AIE are associated with syndromes such as immune dysregulation, polyendocrinopathy, enteropathy, and X-linked inheritance (IPEX) and autoimmune polyendocrinopathy, candidiasis, and ectodermal dystrophy (APECED) [82,83]. Specific autoantibodies, including those targeting a 75 kDa antigen expressed in gut and kidney epithelia, are implicated in enteropathy associated with IPEX syndrome [85]. In APECED syndrome, anti-tryptophan hydroxylase-1 (TPH-1) antibodies are detected in approximately 80% of patients [86]. These antibodies aid in differentiating genetic syndromes. While primarily observed in pediatric populations, adult cases of AIE have been associated with various autoimmune disorders, including type 1 diabetes mellitus, autoimmune thyroiditis, autoimmune hepatitis, rheumatoid arthritis, and common variable immunodeficiency (CVID) [87,88,89,90].

Isolated forms of AIE also occur, diagnosed through the detection of serum anti-enterocyte antibodies (AEA) and anti-goblet cell antibodies (AGA). However, AEA are not specific, being identified in 80–90% of AIE patients and occasionally in individuals with conditions such as HIV, inflammatory bowel disease (IBD), or celiac disease (CD) [82,83]. Similarly, AGA are nonspecific and appear in approximately 28% of patients with CD or IBD [91,92].

Characteristic small bowel histology includes villous blunting, crypt lymphocytosis, and epithelial apoptosis with minimal surface lymphocytosis, distinguishing it from CD (that shows similar degrees of lymphocytosis in the crypt and surface epithelium). The presence of anti-enterocyte or anti-goblet cell antibodies is considered as supportive rather than essential for diagnosis [91]. Notably, antibody titers do not correlate with histologic severity [93].

Histological findings in AIE often overlap with CD, as villous atrophy is a consistent feature. However, intraepithelial lymphocytosis is less common in AIE. Key histological clues include neutrophilic crypt involvement, increased apoptosis, and the absence of goblet and/or Paneth cells in cases associated with anti-goblet cell antibodies [83,87]. The entire gastrointestinal tract can be affected, with some cases demonstrating features of collagenous enteritis or colitis [82,87].

Four histopathological patterns of AIE in 2017 [94] were described as follows:

1. Active chronic enteritis: The most prevalent pattern, marked by moderate-to-severe villous blunting, lamina propria lymphoplasmacytic infiltrates, epithelial apoptosis, and neutrophilic cryptitis or crypt abscesses.
2. CD-like pattern: Characterized by moderate villous blunting and increased intraepithelial lymphocytes (>40 lymphocytes per 100 enterocytes).
3. Graft-versus-host disease (GvHD)-like pattern: Distinguished by prominent crypt epithelial apoptosis with minimal inflammation; villous blunting may also be present.
4. Mixed/no predominant pattern: Exhibits features from two or more patterns, including villous blunting, inflammatory infiltrates, and crypt epithelial apoptosis.

A recent Dutch retrospective study involving 13 patients (the second largest case series) confirmed that active chronic enteritis was the most common histological pattern (62%), followed by CD-like (23%) and GvHD-like (15%) presentations. Notably, two patients had been initially diagnosed with CD. Histological examination in these cases revealed villous atrophy with active chronic enteritis and apoptotic bodies. Among the 13 patients, 11 without history of CD carried CD-associated HLA genotypes. Although CD-specific antibodies were negative, these patients underwent a gluten-free diet (GFD) trial to exclude seronegative CD [95].

### 3.8. Drug-Induced Enteropathy

A comprehensive assessment of a patient’s medication regimen at the onset of diarrhea is crucial for identifying cases of drug-induced diarrhea. If a potential causative agent is identified, it should be discontinued during the trial period. Symptom resolution following discontinuation supports the diagnosis, and the drug should be permanently withdrawn. In instances where the diarrhea is mild, and the medication is essential, a cautious re-challenge may be considered. Chronic diarrhea associated with weight loss presents additional diagnostic challenges, particularly in the absence of a clear temporal association with the initiation of medication. Specific drug-induced conditions, such as olmesartan-associated enteropathy (OAE) and mycophenolate mofetil (MMF)-induced villous atrophy, can be confirmed through small intestine biopsy.

**Nonsteroidal anti-inflammatory drugs (NSAIDs)** are a common cause of duodenitis but may also involve the distal ileum or colon. While duodenitis induced by NSAIDs does not typically present with symptoms characteristic of celiac disease (CD), the histological findings can mimic CD. NSAID-related duodenal changes may include an increased number of intraepithelial lymphocytes (IELs) alongside nonspecific inflammation of the lamina propria involving neutrophils and plasma cells. However, villous atrophy is not typically observed. Additional mucosal alterations associated with NSAID use include erosions, ulcers, and foveolar metaplasia [96].

**Olmesartan-induced enteropathy.** Olmesartan, an antihypertensive medication, belongs to the class of angiotensin II receptor blockers (ARBs). It is the most notable agent in its class associated with enteropathy, although rare cases have also been reported with other ARBs, including irbesartan, valsartan, telmisartan, and eprosartan [97,98,99,100]. This causal link was first identified in 2012 by Rubio-Tapia et al. [101] and subsequently corroborated by a French study [97]. Olmesartan-associated enteropathy (OAE) predominantly affects individuals in their seventh or eighth decade of life, consistent with the age demographic most commonly affected by hypertension [102].

Histopathological findings in OAE are often indistinguishable from those of celiac disease (CD), typically demonstrating villous atrophy. However, the intraepithelial lymphocyte (IEL) count may vary, ranging from normal to increased [102]. Additionally, OAE may mimic autoimmune enteropathy (AIE) by exhibiting features such as loss of Paneth and goblet cells and crypt apoptosis [103]. Distinguishing characteristics include prominent infiltration of the lamina propria by neutrophils and lymphocytes or the presence of a thickened collagenous subepithelial band, which resembles collagenous sprue and aids in differentiating OAE from CD. Importantly, cessation of olmesartan leads to clinical and histological improvement, typically within 3 to 12 months [101].

**Mycophenolate mofetil (MMF)** is an immunosuppressive agent commonly used to prevent graft rejection following organ transplantation. Its histological effects can mimic those observed in graft-versus-host disease (GvHD). MMF-induced enteropathy is characterized by villous blunting, although intraepithelial lymphocytosis (IEL) is generally absent [104]. A distinguishing feature favoring MMF-induced changes is the presence of eosinophils, exceeding 15 eosinophils per 10 high-power fields, whereas findings such as endocrine cell aggregates and hypereosinophilic crypt degeneration are more indicative of GvHD.

Additional histopathological changes associated with MMF use include crypt architectural disarray, chronic inflammation in lamina propria, increased crypt apoptosis, and cystic dilation of duodenal crypts. Clinical symptoms, particularly diarrhea, typically resolve completely following cessation of MMF therapy [105].

**Immune Checkpoint Inhibitors (ICIs)—Ipilimumab** are monoclonal antibodies targeting cytotoxic T-lymphocyte antigen 4 (CTLA-4), belonging to a class of immune checkpoint inhibitors (ICIs) used in cancer therapy for conditions such as metastatic melanoma, ovarian cancer, renal cell carcinoma, and prostate cancer. By antagonizing inhibitory immune pathways, ipilimumab enhances immune-mediated antitumor responses. However, as this mechanism is not tumor-specific, it frequently elicits immune responses in non-cancerous tissues. In addition to ipilimumab, immune-related enteropathy has also been reported with PD-1/PD-L1 inhibitors such as nivolumab and pembrolizumab, which are now widely used in oncologic practice. These agents can produce histological features similar to CTLA-4 blockade, including villous atrophy, increased intraepithelial lymphocytes, and lamina propria inflammation.

Diarrhea is the second most common adverse effect of ipilimumab, following dermatologic manifestations, and represents the most frequent reason for discontinuation of ICI therapy [106,107]. Immune checkpoint inhibitor-induced enterocolitis should be considered in all patients presenting with gastrointestinal symptoms after receiving at least one dose of an ICI, even long after the drug has been discontinued [108].

ICI-associated enteropathy may involve both the small intestine and the colon, with overlapping but distinct histological patterns. In the small intestine, common findings include villous blunting or atrophy, increased IELs, crypt epithelial apoptosis, and lamina propria expansion with a mixed inflammatory infiltrate. These changes can resemble other immune-mediated conditions such as celiac disease, autoimmune enteropathy, or graft-versus-host disease (GVHD). In contrast, in the colon, ICI-related colitis often presents cryptitis and crypt abscesses, increased epithelial apoptosis, especially in the crypt base, surface epithelial injury and mucosal ulceration in more severe cases, occasional features mimicking ulcerative colitis, Crohn’s disease, or microscopic colitis. In some cases, a GVHD-like pattern, with crypt dropout and sparse lamina propria inflammation. Understanding these site-specific patterns is crucial for accurate diagnosis, particularly when histologic overlaps exist with other inflammatory or autoimmune GI disorders. Diagnosis is aided by correlating histologic features with clinical history, immunotherapy exposure, and symptom distribution.

Although diarrhea associated with the ICI use typically results from colitis, which more commonly affects the left colon [109,110,111], ileal involvement has also been reported. In one study, 6% of patients with colitis were found to have ileal involvement; however, the extent of ileal intubation and whether the ileitis occurred in isolation or concurrently with colitis was not specified [110]. Another study identified ileal involvement in 20% of patients undergoing colonoscopy [112].

The same group reported that checkpoint inhibitor therapy may lead to chronic duodenitis, characterized by patchy villous atrophy and increased lamina propria eosinophils, though intraepithelial lymphocytes (IELs) were not assessed [112]. A separate investigation of upper gastrointestinal tract inflammation reported gastritis in 40% and duodenitis in 18% of cases. Duodenal biopsies revealed chronic lymphocytic inflammation, increased IELs, and villous atrophy [113].

In 2019, Zhang et al. reviewed biopsies from 39 patients with suspected ICI-induced enteropathy. In the duodenum, they observed villous blunting, a diffuse increase in lamina propria lymphocytes and plasma cells, and, in some cases, non-necrotizing granulomas. Among patients with villous atrophy, half exhibited a patchy increase in IELs, exceeding the normal threshold of ≤20 lymphocytes per 100 epithelial cells [114].

Ipilimumab is well-documented to cause enterocolitis as a common gastrointestinal side effect. Notably, there is a reported case of ipilimumab inducing severe new-onset celiac disease (CD), suggesting that it may unmask latent CD [115,116].

### 3.9. Small Intestinal Bacterial Overgrowth (SIBO)

SIBO is characterized by an increase in the number and/or alteration in the composition of bacteria within the upper gastrointestinal tract. The gold standard for diagnosing SIBO is the detection of ≥10^5^ colony-forming units per milliliter of fluid obtained from proximal jejunal aspiration [117]. However, a less invasive diagnostic alternative is the hydrogen breath test [118].

SIBO may arise from various underlying conditions, including post-surgical anatomical alterations that cause stasis of intestinal contents (e.g., blind loop syndrome), motility disorders (e.g., systemic sclerosis, neuromuscular disorders, diabetes mellitus), and chronic diseases involving the liver, kidneys, or pancreas [117,118].

The bacterial overgrowth associated with SIBO can impair nutrient absorption by damaging the intestinal brush border. Histological findings in SIBO are non-specific and may range from entirely normal to abnormal. Lappinga et al. identified villous atrophy in 16 out of 67 small bowel biopsies from SIBO patients and reported increased intraepithelial lymphocytes (IELs) in 22 cases [119]. They also noted a potential increase in intraepithelial neutrophils and lymphocytes. To conclude, histological findings in SIBO may include increased intraepithelial lymphocytes with preserved villous architecture, a pattern also seen in Marsh 1 lesions. However, such findings are non-specific and not diagnostic of celiac disease, underscoring the importance of clinical and serologic correlation in differentiating these entities.

SIBO typically responds well to antibiotic therapy, which aids in restoring a balanced gut microbiome.

### 3.10. Inflammatory Bowel Disease

Crohn’s disease is a form of inflammatory bowel disease (IBD) that can involve any segment of the gastrointestinal tract, from the oral cavity to the anus. The terminal ileum is the most commonly affected site, with involvement of the proximal small intestine observed in only 5% of cases [120]. Due to its characteristic transmural inflammation, Crohn’s disease can lead to complications, such as ulcerations, stenosis, fistulae, and abscess formation.

The differential diagnosis for Crohn’s disease includes other forms of IBD, celiac disease, or gastrointestinal malignancies. In the duodenum, it typically manifests as duodenitis accompanied by architectural distortion [121,122]. Histological findings include cryptitis, crypt abscesses, neutrophilic infiltration, and crypt distortion characterized by shortening and branching. A key histological feature in the diagnosis of Crohn’s disease is the presence of non-caseating granulomas, which are usually small, poorly formed collections of epithelioid histiocytes in the lamina propria, often without central necrosis. While granulomas are considered highly specific for Crohn’s disease, particularly in distinguishing it from ulcerative colitis, their detection rate varies between 15% and 36% in surgical and biopsy specimens. The variability is largely due to sampling limitations, disease heterogeneity, and histologic processing. Although granulomas are not required for a diagnosis of Crohn’s disease, their identification significantly increases specificity, especially when infectious causes (e.g., tuberculosis and Yersinia) have been excluded. However, because of their relatively low sensitivity, the absence of granulomas does not rule out Crohn’s disease. To increase the likelihood of granuloma detection, it is recommended that multiple biopsy samples be obtained from both involved and uninvolved areas of the bowel. Detection rates are higher when deeper tissue levels (e.g., submucosa or lymphoid aggregates) are sampled, and particularly in the terminal ileum or in pediatric patients with early disease onset [123,124].

It is essential to distinguish duodenal Crohn’s disease from other causes of injury, such as peptic or medication-induced damage, which can present with overlapping histological features.

Intestinal tuberculosis (ITB) is another critical differential diagnosis in patients presenting with chronic small bowel inflammation and granulomatous lesions, especially in regions where tuberculosis is endemic. ITB can involve the small intestine, particularly the terminal ileum, and may present with symptoms and histologic features that closely mimic Crohn’s disease, including ulceration, strictures, and granulomatous inflammation. Both Crohn’s disease and intestinal tuberculosis can present with segmental small bowel involvement, ulcers, strictures, and granulomas. However, several features can favor a diagnosis of ITB: granulomas in ITB tend to be larger, more confluent, and often caseating, whereas in Crohn’s disease they are usually small, scattered, and non-caseating; acid-fast bacilli (AFB) may be identified on Ziehl–Neelsen staining in ITB, though sensitivity is low; PCR for Mycobacterium tuberculosis, culture, and response to anti-TB therapy support the diagnosis; and clinically, systemic symptoms (fever, night sweats, weight loss) and pulmonary involvement may also favor ITB. Recognizing these features is essential for avoiding misdiagnosis, particularly in endemic areas or immunocompromised patients [125,126].

### 3.11. Eosinophilic Gastro-Enteritis (EGE)

Idiopathic eosinophilic gastroenteritis (EGE) is a diagnosis of exclusion characterized by eosinophilic infiltration of the gastrointestinal tract in the absence of identifiable secondary causes, such as medication-induced injury, parasitic infections, Crohn’s disease, collagen vascular disorders, or hematologic malignancies. The infiltration may involve the mucosal, submucosal, or serosal layers of the gastrointestinal wall, with clinical manifestations varying according to the layer involved [127].

In cases where the mucosal layer is affected, bowel wall thickening can lead to symptoms of obstruction. Combined mucosal and submucosal involvement typically presents with abdominal pain, nausea, vomiting, diarrhea, malabsorption, and weight loss [128]. When the serosal layer is involved, ascites may develop as a prominent feature [129].

Histological examination of mucosal involvement in EGE may reveal variable degrees of villous shortening, crypt hyperplasia, and increased IELs. The eosinophilic inflammatory infiltrate is a hallmark finding and may include degranulated eosinophils, intraepithelial eosinophils, or eosinophilic crypt abscesses.

### 3.12. Graft-Versus-Host Disease (GVHD)

GVHD involving the gastrointestinal tract is a recognized complication of bone marrow transplantation, though it can also occur, albeit rarely, following solid organ transplantation or blood transfusion [130]. Clinical manifestations range from mild symptoms such as nausea, vomiting, and diarrhea to severe complications, including gastrointestinal bleeding, protein-losing enteropathy, and ileus.

Histopathological findings in gastrointestinal GVHD develop progressively through the following stages:

1. Isolated apoptotic epithelial cells without crypt damage;
2. Apoptosis accompanied by the loss of individual crypts;
3. Apoptosis with the destruction of two or more adjacent crypts, with or without the formation of apoptotic crypt abscesses;
4. Extensive crypt loss, leading to mucosal denudation and replacement by granulation tissue.

Across all stages, inflammatory infiltrates are typically sparse [131]. A hallmark of intestinal GVHD is the targeting of Paneth cells, with the extent of Paneth cell loss correlating directly with the severity of the disease [132].

## 4. Conclusions

The spectrum of immune-mediated enteropathies has broadened significantly, presenting considerable diagnostic challenges due to the overlapping clinical features. When clinical symptoms alone are insufficient for a definitive diagnosis, histological evaluation serves as an indispensable diagnostic modality. However, pathognomonic histological features for specific drug-induced etiologies are rare, underscoring the critical importance of effective collaboration between gastroenterologists and pathologists. Examination of the duodenal mucosa is not only instrumental in diagnosing these conditions but also essential for assessing disease progression, evaluating therapeutic responses, and identifying potential complications. This review seeks to elucidate the subtle histopathological distinctions among various enteropathies (Table 3 and Table 4).

## Figures and Tables

**Table 1:** Histologic differential diagnosis based on pattern.

Normal Villous Architecture and Increased IELs	Villous Atrophy with/Without Increased IELs
Early developing celiac disease (Marsh I–II)	Overt celiac disease (Marsh III a–c), and complicated celiac disease (e.g., refractory sprue, collagenous sprue)
Helicobacter pylori-associated gastroduodenitis	Collagenous sprue
Drugs: NSAIDs, selective serotonin reuptake inhibitors, proton pump inhibitor	Drugs: mycophenolate mofetil, colchicine, Angiotensin receptor blocker, monoclonal antibodies
Tropical sprue	Autoimmune enteropathy
Infections: viral enteritis, Giardia, Cryptosporidium	Immunodeficiency: common variable immune deficiency
Immune conditions: rheumatoid arthritis, Hashimoto thyroiditis, systemic lupus erythematosus, autoimmune enteropathy	Graft-versus-host disease
Immunodeficiency: common variable immune deficiency	Inflammatory bowel disease
Graft-versus-host disease	Chemoradiation therapy
Inflammatory bowel disease	Eosinophilic enteritis
Small intestinal bacterial overgrowth	Infection: Tropical sprue, Tuberculosis, Whipple disease, Giardiasis, Acquired immune deficiency syndrome enteropathy
Irritable bowel syndrome	Neoplasia: systemic mastocytosis, T-cell lymphoma

**Table 2:** Comparison of the **Marsh–Oberhuber** and **Corazza–Villanacci** classification systems for celiac disease.

Name	Marsh–Oberhuber	Corazza–Villanacci
**Main Use**	Celiac disease histological grading	Simplified alternative for routine pathology
**Histologic Features**	IELs, crypt hyperplasia, villous atrophy	IELs, crypt hyperplasia, villous atrophy
**Grading Structure**	Types 0–3c (0 = normal, 3c = total atrophy)	Grade A: increased IELs only; Grade B1: partial atrophy; B2: total atrophy
**Key Diagnostic Threshold**	>25–30 IELs/100 enterocytes + crypt/villous changes	>25 IELs/100 enterocytes
**Strengths**	Detailed morphological staging; widely used	Simplified, improved reproducibility
**Limitations**	Greater interobserver variability; complex for routine use	Less granular detail; not ideal for research trials

**Table 3:** Summary of diagnostic criteria for major enteropathies.

Condition	Diagnostic Criteria
**Celiac disease (CD)**	Symptoms (e.g., diarrhea and weight loss) or screening in risk groups (type 1 diabetes, first degree relatives) Positive serology (e.g., anti-tTG, EMA) Histology: >25 IELs/100 enterocytes, villous atrophy (Marsh ≥ 2) Response to gluten-free diet (GFD) HLA-DQ2/DQ8 (negative predictive value in inconclusive cases) (ACG 2023, ESPGHAN)
**Refractory celiac disease (RCD)**	Persistent symptoms and villous atrophy after ≥12 months of strict GFD Negative celiac serology Confirmation of GFD adherence (dietitian) Histology: severe atrophy, crypt apoptosis RCD II: clonal IELs lacking surface CD3/CD8 (Mayo Clinic, BSG)
**Common variable immunodeficiency (CVID) enteropathy**	Hypogammaglobulinemia (low IgG, IgA, IgM) Chronic diarrhea and recurrent infections Histology: increased IELs, villous atrophy, absent plasma cells Serology often negative for CeD due to antibody deficiency
**Autoimmune enteropathy (AIE)**	Chronic diarrhea and weight loss Presence of anti-enterocyte or anti-goblet cell antibodies (supportive) Histology: villous blunting, crypt apoptosis, low or absent goblet/Paneth cells Exclusion of other causes Often associated with autoimmune disorders
**Olmesartan-associated enteropathy**	History of olmesartan or related ARB use Chronic diarrhea, weight loss Histology: villous atrophy, increased IELs (variable), crypt apoptosis, collagen band Resolution after drug withdrawal Negative CeD serology
**Graft-versus-host disease (GVHD)**	History of stem cell or organ transplant GI symptoms (e.g., diarrhea, pain, bleeding) Histology: apoptotic bodies in crypts, crypt loss, mucosal denudation Sparse inflammation Paneth cell loss correlates with severity

**Table 4:** Main histopathological findings.

Condition	IELs	Villous Atrophy	Other Histological Features
**Celiac Disease**	↑ (>25–30/100 ECs)	Partial to total (Marsh)	Crypt hyperplasia, lamina propria inflammation, CD3+/CD8+ T-cells
**Refractory CD (Type II)**	↑ (clonal, aberrant)	Severe	Crypt apoptosis, subepithelial collagen, risk of EATL
**Autoimmune Enteropathy**	Variable or ↓	Yes	Apoptosis, cryptitis, loss of goblet/Paneth cells, AEA/AGA antibodies
**CVID Enteropathy**	Normal to ↑	Variable	Plasma cell depletion, lymphoid hyperplasia, apoptosis
**Olmesartan Enteropathy**	Normal or ↑	Yes	Crypt apoptosis, collagen band, mimics AIE or CD
**Tropical Sprue**	↑ (crypts/basal villi)	Mild-moderate	Eosinophilia, patchy involvement, entire small intestine affected
**SIBO**	Mild ↑ or normal	Often none	Nonspecific: increased IELs, occasional neutrophils, mild changes
**GVHD**	Minimal or variable	Yes	Apoptotic bodies, crypt dropout, Paneth cell loss, sparse inflammation
**Collagenous Sprue**	Variable (↑ or normal)	Yes	Thick subepithelial collagen band (>10 µm), inflammation
**Crohn’s Disease**	Variable	Sometimes	Cryptitis, granulomas (non-caseating), distortion, transmural inflammation
**Eosinophilic Enteritis**	↑ ± eosinophils	Variable	Eosinophilic infiltration, crypt abscesses, degranulated eosinophils
**Drug-Induced Enteropathy**	Normal to ↑	Possible	Variable: increased IELs, eosinophils, apoptosis, crypt disarray

↑ (above normal value); ↓ (less than normal value).

## Data Availability

No new data were created or analyzed in this study. Data sharing is not applicable to this article.
